# Effects of strict containment policies on COVID-19 pandemic crisis: lessons to cope with next pandemic impacts

**DOI:** 10.1007/s11356-022-22024-w

**Published:** 2022-08-04

**Authors:** Mario Coccia

**Affiliations:** CNR–National Research Council of Italy, Collegio Carlo Alberto, Via Real Collegio, 30, Moncalieri, 10024 Turin, Italy

**Keywords:** COVID-19 pandemic, Non-pharmaceutical interventions, Containment policy, Health policy, Stringency index, Infections, Fatality rates, Medical ventilation, New technology

## Abstract

The goal of the study here is to analyze and assess whether strict containment policies to cope with Coronavirus Disease 2019 (COVID-19) pandemic crisis are effective interventions to reduce high numbers of infections and deaths. A homogenous sample of 31 countries is categorized in two sets: countries with high or low strictness of public policy to cope with COVID-19 pandemic crisis. The findings here suggest that countries with a low intensity of strictness have average confirmed cases and fatality rates related to COVID-19 *lower *than countries with high strictness in containment policies (confirmed cases are 24.69% *vs.* 26.06% and fatality rates are 74.33% *vs. *76.38%, respectively, in countries with low and high strictness of COVID-19 public policies of containment). What this study adds is that *high levels* of strict restriction policies may not be useful measures of control in containing the spread and negative impact of pandemics similar to COVID-19 and additionally a high strictness in containment policies generates substantial social and economic costs. These findings can be explained with manifold socioeconomic and environmental factors that support transmission dynamics and circulation of COVID-19 pandemic. Hence, high levels of strictness in public policy (and also a high share of administering new vaccines) seem to have low effectiveness to stop pandemics similar to COVID-19 driven by mutant viral agents. These results here suggest that the design of effective health policies for prevention and preparedness of future pandemics should be underpinned in a good governance of countries and adoption of new technology, rather than strict and generalized health polices having ambiguous effects of containment in society.

## Introduction

Public policies of countries to cope with infectious diseases similar to Coronavirus Disease 2019 (COVID-19) can have different levels of strictness to lower negative pandemic impact in terms of high numbers of infections and deaths (Anttiroiko 2021; Bontempi et al. 2021; Bontempi and Coccia 2021; Coccia 2022a; Nicoll and Coulombier 2009; Vinceti et al. 2021). In particular, government responses of countries to cope with COVID-19 can have a high degree of strictness, such as a long period of full lockdown and quarantine, general travel bans at domestic and international level, compulsory facemask coverings indoors and outdoors, and widespread impositions to circulation of people that reduce public and private life in society, etc. (Allen 2022; Askitas et al. 2021; Kim and Lee 2022; Wieland 2020). The strictness of policy responses to face COVID-19 pandemic crisis can be measured with a combination of different indicators that are aggregated in the stringency index, which is processed by the Oxford Coronavirus Government Response Tracker project (Hale et al. 2021; Stringency Index 2022). Mahmoudi and Xiong (2022) argue that lower COVID-19 infections and mortality rates are associated with stricter enforcement policies and more severe penalties for violating stay-at-home orders and other control measures. Qiu et al. (2022) point out that bans of travel, closing schools and economic activities, and other restrictions were found to be the most effective responses to reduce COVID-19 transmission. However, an unknown question is what containment policies to cope with COVID-19 crisis are more effective measures: i.e., if the appropriate strategy is the design and implementation of a public poliy with a high *or* low degree of restrictions in society (Wood 2021). The goal of the study here is to confront this vital question with the evaluation of the effectiveness of different public policies to cope with COVID-19 pandemic crisis in reducing infections and deaths and also in sustaining economic growth. A comparative analysis between countries that introduced a high degree of restrictions and countries with a low strictness of COVID-19 containment policies can clarify the effects of interventions in society. In fact, the identification and understanding of the effects of different types of containment policies provide critical aspects for planning and improving effective responses of crisis management to cope with next pandemic impacts, similar to COVID-19, in socioeconomic system and total environment (Barro 2020; Coccia 2021a, b, 2022b, c, d).

## Methods

### Sample

The sample is based on 31 countries belonging to the Organisation for Economic Co-operation and Development (OECD Data 2022a) with a Gross Domestic Product (GDP) per capita higher than U$16,000 to have a homogenous framework for statistical analyses. Countries of the sample are: Australia, Austria, Belgium, Canada, Czech Republic, Denmark, Estonia, Finland, France, Germany, Greece, Hungary, Iceland, Ireland, Israel, Italy, Japan, Latvia, Lithuania, Netherlands, New Zealand, Norway, Poland, Portugal, Slovakia, Slovenia, Spain, Sweden, Switzerland, UK, and USA.

### Measures for statistical analyses

- Strictness of COVID-19 health policies in nations is measured with the stringency index that aggregates different indicators of government responses to face COVID-19 pandemic crisis (e.g., period of school closure, business closure, quarantine, travel reduction, cancellation of events, and orders for vaccination policies). Minimum of the index = 0, maximum = 100, which indicates the highest strictness of public policy to cope with COVID-19 pandemic crisis. Period under study: January 2020–January 2022 (Hale et al. 2021; Stringency Index 2022).
- Wealth of nations is measured with GDP per capita in 2020, constant 2010 US$ (The World Bank 2022a).
- Economic growth is measured with GDP (annual) growth rate % in 2020 (OECD Data 2022b).
- Health expenditure (% of GDP) over 2008–2018, last period available (The Word Bank 2022a).
- Population total of the year 2020 (The World Bank 2022c).
- COVID-19 vaccination is measured with the percent share of people fully vaccinated against COVID-19 in countries on 14 February 2022 (Our World in Data 2022a).
- COVID-19 infected individuals (%) are the percent ratio between confirmed cases of COVID-19 (on 21 February 2022) and population (Johns Hopkins Center for System Science and Engineering 2022).
- Mortality related to COVID-19 is measured with Case Fatality Ratio (CFR) % on 21 February 2022 (cf., Coccia 2021b; WHO 2020; Wilson et al. 2020):

$$\mathrm{Case}\;\mathrm{Fatality}\;\mathrm{Ratio}\;\left(\mathrm{CFR}\right)\%=\left(\frac{\mathrm{Number}\;\mathrm{of}\;\mathrm{deaths}\;\mathrm{from}\;\mathrm{COVID}-19}{\mathrm{Number}\;\mathrm{of}\;\mathrm{confirmed}\;\mathrm{cases}\;\mathrm{of}\;\mathrm{COVID}-19}\right)\times100$$

Source of data: Johns Hopkins Center for System Science and Engineering (2022).

- Medical ventilators are the number of these technological devices per 100,000 inhabitants over 2015–2020 period, last period available (Our World in Data 2022b).

### Data analysis procedure 

**Fig. 1:**
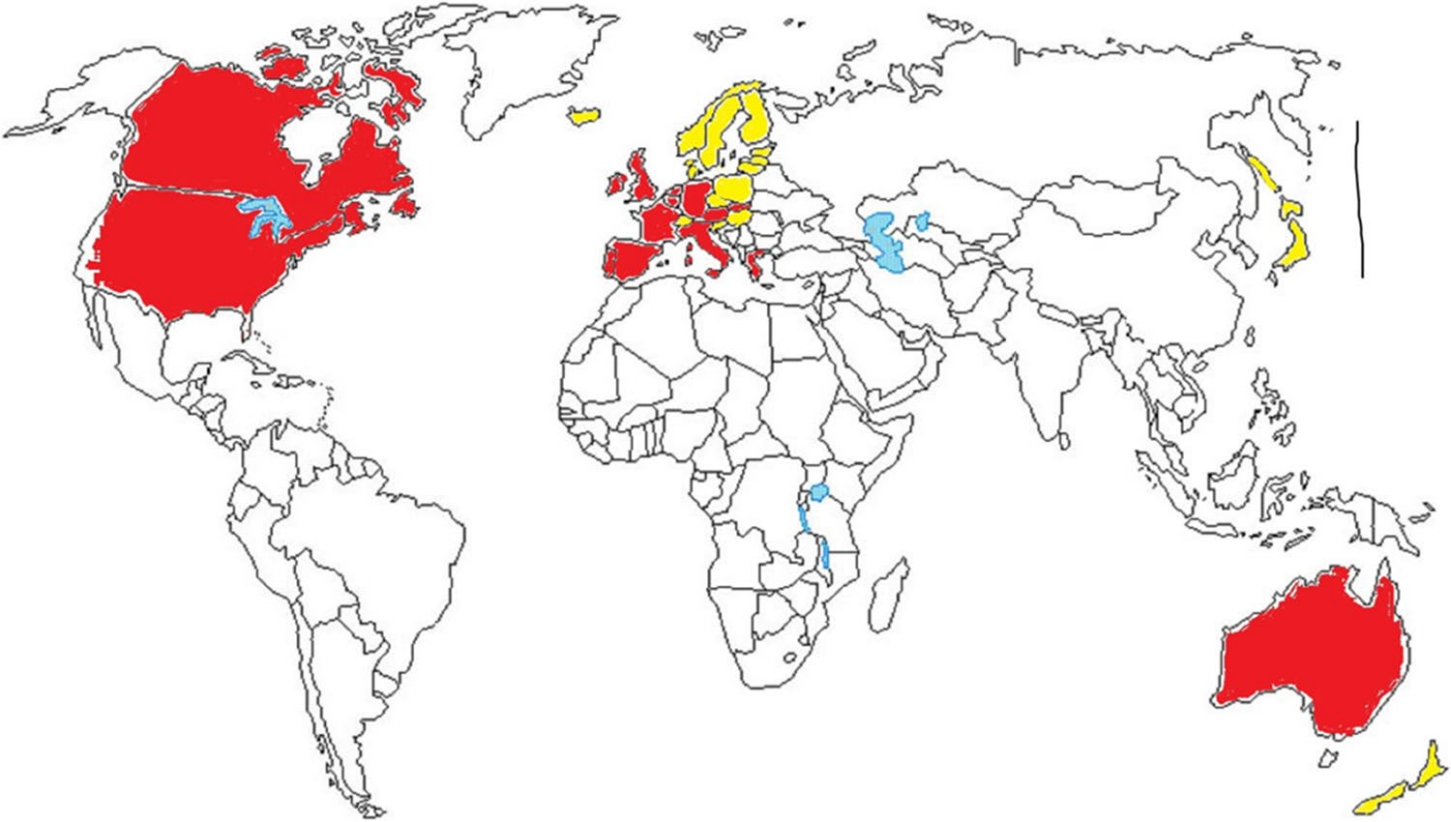
Map of countries with a high and low strictness in policy responses to cope with COVID-19 pandemic. Countries with *high* strict policies of containment have red color: Australia, Austria, Belgium, Canada, France, Germany, Greece, Ireland, Israel, Italy, Netherlands, Portugal, Slovakia, Spain, UK, and USA. Countries with *low* strictness in containment policies have a color yellow in the map: Czech Republic, Denmark, Estonia, Finland, Hungary, Iceland, Japan, Latvia, Lithuania, New Zealand, Norway, Poland, Slovenia, Sweden, and Switzerland

*Firstly*, the containment (stringency) index of countries under study is used to categorize the sample in two sets:

- **Group 1**: Countries with a *high degree* of restrictions and mandatory measures to cope with COVID-19 pandemic crisis: average containment index over 2020–2022 period is higher than 50 points (100 = max of restrictions). These countries have introduced a lot of non-pharmaceutical measures for pandemic control that are characterized by a long period of full lockdown (including school closure, workplace and business closure, and a longer average period of quarantine), widespread domestic and international travel reduction, compulsory wearing face masks outdoors and indoors, and cancellation of all public and private events (Coccia 2021c, d). Moreover, some countries have also introduced mandates for vaccinations of people working in specific and/or all economic sectors (Coccia 2021e, 2022d, e). These countries are: Australia, Austria, Belgium, Canada, France, Germany, Greece, Ireland, Israel, Italy, Netherlands, Portugal, Slovakia, Spain, UK, and USA (Fig. 1).
- **Group 2**: Countries with a low degree of restrictions and impositions to face COVID-19 pandemic crisis: average containment (stringency) index has a score lower than 50 points. In general, these countries have introduced few non-pharmaceutical measures of pandemic control, and having a short duration moreover, vaccination policies in these countries are based on incentives and not compulsory rules (Coccia 2022e). These countries are: Czech Republic, Denmark, Estonia, Finland, Hungary, Iceland, Japan, Latvia, Lithuania, New Zealand, Norway, Poland, Slovenia, Sweden, and Switzerland (Fig. 1).

*Remark*: This study considers OECD countries for which all data of variables under study are available, whereas other countries like China (that appropriately controlled the new coronavirus --SARS-Cov 2-- and apply strict policies for stopping the spread of COVID-19 in cities) are not included because data of some variables are missing, and all statistical analyses cannot be performed.

*Secondly*, the bivariate and partial correlations (controlling health expenditure as % of GDP) assess all associations of variables under study. The follow-up investigation is a comparative analysis of the two groups using descriptive statistics (cf., Coccia 2018a). Moreover, independent samples *T*-test analyzes if the arithmetic means of variables between groups 1 and 2 (above) are significantly different and, as a consequence, if countries with a high level of strictness in containment policy to cope with COVID-19 pandemic crisis, they effectively reduce infections and deaths and sustain economic growth.

## Results

Results show a positive (and significant) association between containment (stringency) index and fatality rate (*r* = 0.34, *p*-value 0.05), and full vaccinated people (*r* = 0.50, *p*-value 0.01), whereas correlation is negative with GDP growth (annual %). These results are confirmed with partial correlation, controlling average health expenditure (Table 1).

**Table 1:** Correlation analysis

*Bivariate correlation*	Log average containment index 2020–2022	Log full vaccinated people February 2022	Log confirmed cases 21 February 2022	Log fatality rate 21 February 2022	GDP growth (annual %), 2020
Log average containment index 2020–2022	1	0.496**	0.263	0.336*	− 0.324*
*Partial correlation* Control variable: Log average health expenditure 2008–2018	Log average containment index 2020–2022	Log full vaccinated people February 2022	Log confirmed cases 21 February 2022	Log fatality rate 21 February 2022	GDP growth (annual %), 2020
Log average containment index 2020–2022	1	0.465	0.289	0.381	− 0.300
Significance (1-tailed)		0.006	0.064	0.021	0.057

*Note *1. **Correlation is significant at the 0.01 level (1-tailed), *correlation is significant at the 0.05 level (1-tailed). *Note 2*: Control variable log average health expenditure 2008–2018

The categorization of countries is visualized geographically in Fig. 1:

- *Countries with a low* level of control measures in containment policies to face COVID-19 have a stringency index over 2020–2022 (January) period equal to an average value of 47.8 points (std. error 0.99). Countries with a yellow color in Fig. 1.
- *Countries with a high* level of control measures in containment policies *(high strictness)* have a mean index of stringency over 2020–2022 (January) period = 59.6 points (std. error 1.05). Countries with a red color in the map (Fig. 1).

**Fig. 2:**
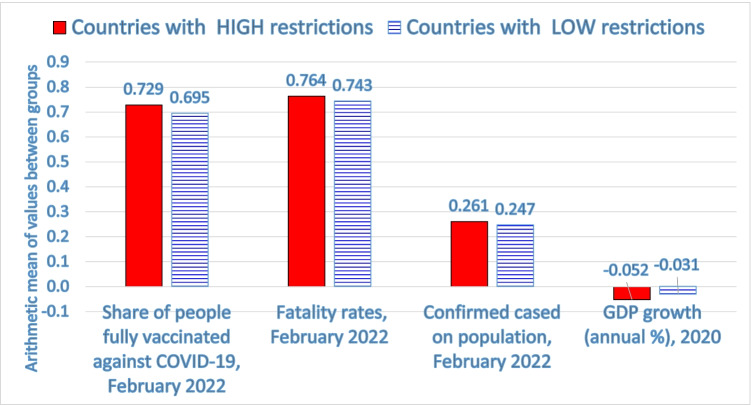
Comparative analysis of health and economic indicators between countries with a high and low strictness in restrictions to cope with COVID-19 pandemic crisis (cf., Table 2 for significance of differences)

Table 2 shows that countries with a high strictness in restrictions in society (average containment index of about 60) have a high share of vaccinations but confirmed cases on population (%) and case fatality rates (%) are higher than countries with a low strictness of COVID-19 containment policies (confirmed cases are 26.06% *vs. *24.69% and fatality rates are 76.38% *vs.* 74.33%, respectively, in countries with high *vs. *low strictness). Comparative analysis of these two groups of countries also shows that average health expenditure (% of GDP) is 8.58% *vs.* 9.8% in countries with low *vs.* high levels of restrictions. In addition, GDP growth (annual %) in 2020 of countries with a high score of restrictions is − 5.2%, which indicates a higher reduction (i.e., a lower economic growth) than countries with a low degree of restrictions and strictness of policy responses to face COVID-19 crisis (latter countries have GDP growth annual = − 3.1%). *T-*test for equality of means suggests a significant difference of the arithmetic means between groups 1 and 2 of countries with high and low degree of restrictions for values of full vaccinated people (*p*-value 0.01), whereas the difference of GDP growth (annual %) in groups 1 and 2 has a low significance (*p*-value = 0.1). Other variables, such as fatality rates and confirmed cases, have not a significant difference (Table 1). Hence, this result suggests that the average level of these variables between groups 1 and 2 has similar values; as a consequence, the introduction of a strategy of high restrictions to reduce infections and deaths related to COVID-19 is an ineffective health policy of containment but certainly it deteriorates economic growth of nations. Figure 2 shows that countries with a high strictness in restrictions have a high negative impact of pandemic in society (i.e., high numbers of confirmed cases and fatality rates) and a high decline of economic growth, though a higher share of vaccinations. In general, these findings reveal that a high strictness of control measures can block the operation of socioeconomic systems without reducing negative effects of COVID-19 pandemic crisis (cf., Allen 2022). Overall, then, policy responses of high restrictions and compulsory measures seem to be *ineffective* to cope with COVID-19 pandemic and mitigate high numbers of infections and deaths, though a high share of vaccinations.

**Table 2:** Descriptive statistics

	Countries with LOW restrictions		Countries with HIGH restrictions		*T-*test for equality of means
Description of variables	M	Std. error mean	M	Std. error mean	Significance *p*-value
- Containment index over 2020–2022 period	**47.823**	0.987	**59.606**	1.054	0.05
- Current health expenditure % of GDP, 2008–2018	**8.578**	0.490	**9.800**	0.593	not sign.
- Share of people fully vaccinated against COVID-19, February 2022	**69.460**	0.020	**72.856**	0.023	0.01
- Confirmed cases/population (%)	**24.69**	3.42	**26.06**	2.24	not sign.
- Fatality rates %, February 2022	**74.333**	0.177	**76.375**	0.082	not sign.
- GDP growth (annual %), 2020 (§)	** − 3.059**	0.489	**− 5.174**	1.083	0.1

*Note*: M = arithmetic mean, which is in boldface; (§) these data have missing values for some countries; *not sign. *= not significant

## Explanation of results

The statistical evidence above seems in general to show a strictness in public policy does not generate a significant effect of reduction of the COVID-19 pandemic impact and it induces negative effects on socioeconomic systems.

The explanation of these results is that restrictions and mandatory measures to cope with COVID-19 pandemic can be *a necessary but not a sufficient strategy* to reduce the negative impact of the novel coronavirus in society because there are manifold social, institutional, and environmental factors that support the diffusion of infections and level of mortality of this pandemic (Atkeson 2021; Coccia 2014, 2017a, 2022f; Núñez-Delgado et al. 2021; Pronti and Coccia 2021; Yao et al. 2022). For instance, a high level of international trade in countries can explain the accelerated transmission dynamics and negative impact of the COVID-19 pandemic because trade generates a high socioeconomic interaction between people and, as a consequence, circulation of viral agents (Bontempi, 2022; Bontempi and Coccia 2021; Bontempi et al. 2021). In this context, Jamison et al. (2021) maintain that in Europe, non-pharmaceutical interventions based on incentives produce positive effects to cope with pandemic impact compared to compulsory rules and/or orders that have a smaller benefit–cost ratio (cf., Coccia 2019a).

A complementary explanation of results is based on Peltzman theory (Peltzman 1975). In fact, strict policy responses and vast vaccination campaigns can certainly help to lower the risk of serious effects of COVID-19, but the Peltzman theory suggests that when similar safety measures are implemented in society, people tend to increase their risky behaviors. This social behavior can be due to a lower people’s perception of risk to be infected, and so people take riskier decisions and have risk behavior that increase the widespread of viral agents, especially of new variants that spread more easily, generating high numbers of infections and fatality rates related to COVID-19 (Khandia et al. 2022; Prasad and Jena 2014). Hence, Peltzman theory predicts that strict safety measures for COVID-19 pandemic crisis (e.g., vast containment policies against COVID-19 and compulsory vaccinations) can generate a lower benefit than expectation because strict control measures are offset by increases in risky behavior of people in society (Iyengar et al. 2022).

Barro (2020) also analyzes non-pharmaceutical interventions and mortality in US cities during the pandemic of 1918–1919 period and shows that the estimated effect on total deaths is small. Many studies show that hard restriction policies, such as full lockdowns of longer period, do not significantly reduce the number of confirmed cases and deaths related to COVID-19 (Allen 2022; Homburg 2020; Jamison et al. 2021; Wieland 2020). Zhu and Tan (2022) assess the effectiveness of Hong Kong’s strict border restrictions with mainland China in curbing the transmission of COVID-19 pandemic. Results show that border restriction policy and its further extension are not useful measures in containing the spread of COVID-19 when the viral agent is circulating in society; at the same time, these containment policies increase economic and social costs. In addition, health policies based on a high degree of restrictions create a state of uncertainty that negatively affects overall socioeconomic system, and can reduce investments in capital and human resources, decrease consumer spending, and increase public debt in situation of crisis management (Goolsbee and Syverson 2021; Coccia 2013, 2017b, 2021e; Coccia and Rolfo 2020). In fact, strict policies of containment and contradictory scientific recommendations for COVID-19 pandemic have created confusion in many countries because a lot of initial claims are subsequently proved to be false or misleading (Ball 2021; Kufel et al. 2022).

In general, the containment of COVID-19 pandemic crisis depends not only on the strictness of health policies but also how these health policies are applied in society, such that delayed and poorly targeted regulatory measures can reduce the appropriateness also of the most reasonable policies of crisis management, generating negative effects on social and economic activities (Coccia 2021c). In fact, in contexts of environmental threath, many countries have showed to have a low preparedness of crisis management, applying strict health policies of containment with the hope to reduce the negative impact of COVID-19 pandemic crisis. Results here suggest that a strategy of strict containment policy is ineffective to cope with COVID-19 impact, based on a mutant viral agent, and generates poor effects of reduction of infections and deaths, and additionally, it damages socioeconomic system (Coccia 2021c, 2022d; Chirumbolo et al. 2022; Gupta et al. 2022).

## Conclusions and public policy implications

In the presence of a global pandemic crisis, one of the goals of nations is to mitigate infections and mortality and support economic growth with appropriate public policies (cf., Coccia 2021c, 2022c).

What this study reveals is:

- Uncertain effects of strict policy responses in curbing high numbers of infections and deaths related to COVID-19
- that high restrictions and compulsory measures seem to be ineffective to mitigate high numbers of infections and deaths, though a high share of vaccinations
- that strict restriction policy may generate substantial economic and social costs and may not be a useful response in containing the spread of COVID-19 pandemic driven by circulation of mutant viral agents in society
- that Peltzman theory and other socioeconomic and environmental factors can explain a high pandemic impact of mutant viral agents also in the presence of high strictness of public policies and a high share of vaccination based on compulsory mandates
- that the preparedness of crisis management in countries to cope with pandemic impacts tends to be poor but it can be improved with a good governance and an increased access to new technology (cf., Coccia 2019b, 2022g; Ardito et al. 2021)

**Fig. 3:**
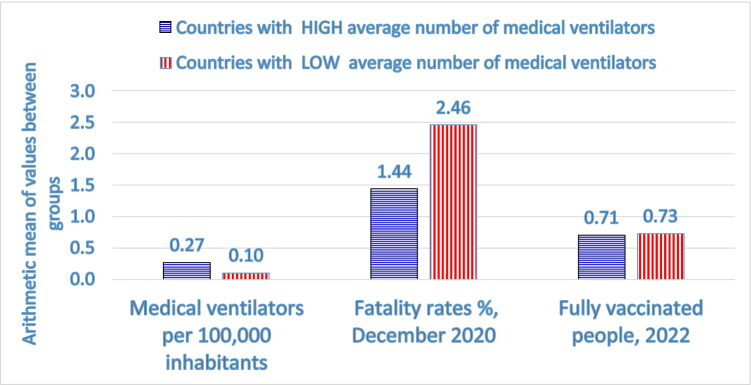
Comparative analysis between countries with a high and low level of medical ventilators per 100,000 people: fatality rate is considered on 31 December 2020, before the COVID-19 vaccination to show the real technological effect of medical ventilators on health system, when this technology was the only approach to treating this new infectious disease because effective drugs lacked

Chirumbolo et al. (2022) argue that the scientific community should support institutions and policymakers to improve best practices of crisis management to face next pandemics. In fact, Benati and Coccia (2022) suggest the positive effects of a good governance in supporting the prompt implementation of health policy responses to cope with pandemic impact, which may mitigate fatality rates. Moreover, an exploratory research based on a small sample of countries shows in Fig. 3 that countries with a high average number of medical ventilators per 100,000 people, they have a low average fatality rate (1.46%), though a lower percent share of people fully vaccinated against COVID-19, compared to countries with a low technological equipment of medical ventilators. Mahmoudi and Xiong (2022) point out that lower COVID-19 mortality rates are linked with an increased access to medical ventilators and intensive care units. Meiry et al. (2022) maintain that the development of medical ventilators to cope with emergency of COVID-19 pandemic, based on a functional rather than a commercial-oriented approach, can support innovations that reduce deaths and a negative pandemic impact in socioeconomic systems (cf. also Coccia and Finardi 2013; Coccia and Bellitto 2018; Coccia 2003; Coccia and Rolfo 2000).

To conclude, the results of this analysis here seem to be that strict health policies (based on many restrictions and obligations of longer duration) do not reduce negative effects of COVID-19 pandemic in society in terms of lower levels of infections and deaths and, additionally, tend to deteriorate social and economic systems. These conclusions are of course tentative. There is need for much more research in these topics because not all confounding factors that affect the policy responses against COVID-19 are considered in this complex inquiry. Results here have also to be reinforced with additional statistical analyses based on a large sample of countries.

Overall, therefore, these findings suggest an *alternative public health policy of crisis management* (cf., Coccia 2018b; 2021e) to face next pandemic crisis, namely: an effective strategy is based on little restrictions, a better communication, and especially a good governance with high levels of investments in health infrastructures and in modern technology of medical ventilators that can really cope with negative effects of future pandemic threats of new viral agents, when effective drugs lack.

## Data Availability

Data derived from public domain resources that are in the references.

## References

[CR1] Allen DW (2022). COVID-19 lockdown cost/benefits: a critical assessment of the literature. Int J Econ Bus.

[CR2] Anttiroiko A-V (2021) Successful government responses to the pandemic: contextualizing national and urban responses to the COVID-19 outbreak in east and west. Int J E-Plan Res (IJEPR), IGI Global 10(2):1–17

[CR3] Ardito L, Coccia M, Messeni Petruzzelli A (2021). Technological exaptation and crisis management: evidence from COVID-19 outbreaks. R&D Manag.

[CR4] Askitas N, Tatsiramos K, Verheyden B (2021) Estimating worldwide effects of non-pharmaceutical interventions on COVID-19 incidence and population mobility patterns using a multiple-event study (Open Access) (2021). Sci Rep 11(1):1972 10.1038/s41598-021-81442-x PMC7820317 33479325

[CR5] Atkeson AG (2021) Behavior and the Dynamics of Epidemics. Brookings Papers on Economic Activity, 67–88. https://www.jstor.org/stable/27093820

[CR6] Ball P (2021) What the COVID-19 pandemic reveals about science, policy and society. Interface Focus 11:20210022. 10.1098/rsfs.2021.0022 10.1098/rsfs.2021.0022 PMC8504882 34956594

[CR7] Barro RJ (2020) Non-pharmaceutical interventions and mortality in U.S. cities during the great influenza pandemic, 1918–1919. NBER Working Paper, No. 27049. 10.3386/w27049 10.1016/j.rie.2022.06.001 PMC9232401 35784011

[CR8] Benati I, Coccia M (2022). Global analysis of timely COVID-19 vaccinations: improving governance to reinforce response policies for pandemic crises. Int J Health Gov.

[CR9] Bontempi E (2022). A global assessment of COVID-19 diffusion based on a single indicator: some considerations about air pollution and COVID-19 spread. Environ Res.

[CR10] Bontempi E, Coccia M (2021) International trade as critical parameter of COVID-19 spread that outclasses demographic, economic, environmental, and pollution factors. Environ Res 201:111514, PII S0013–9351(21)00808–2. 10.1016/j.envres.2021.111514 10.1016/j.envres.2021.111514 PMC8204848 34139222

[CR11] Bontempi E, Coccia M, Vergalli S, Zanoletti A (2021). Can commercial trade represent the main indicator of the COVID-19 diffusion due to human-to-human interactions? A comparative analysis between Italy, France, and Spain. Environ Res.

[CR12] Chirumbolo S, Pandolfi S, Valdenassi L (2022). Seasonality of COVID-19 deaths. Did social restrictions and vaccination actually impact the official reported dynamic of COVID-19 pandemic in Italy. Environ Res.

[CR13] Coccia M (2003) Metrics of R&D performance and management of public research labs. IEMC '03 Proceedings. Managing Technologically Driven Organizations: The Human Side of Innovation and Change, 2003, pp 231–235. 10.1109/IEMC.2003.1252267

[CR14] Coccia M (2013). Employment. Innovation and public debt across economies. Afr J Bus Manag.

[CR15] Coccia M (2014) Steel market and global trends of leading geo-economic players. Int J Trade Glob Mark 7(1):36–52. 10.1504/IJTGM.2014.058714

[CR16] Coccia M (2017). New directions in measurement of economic growth, development and under development. J Econ Pol Econ.

[CR17] Coccia M (2017). Asymmetric paths of public debts and of general government deficits across countries within and outside the European monetary unification and economic policy of debt dissolution. J Econ Asymmetries.

[CR18] Coccia M (2018). An introduction to the methods of inquiry in social sciences. J Soc Adm Sci.

[CR19] Coccia M (2018). An introduction to the theories of institutional change. J Econ Libr.

[CR20] Coccia M (2019a) Comparative incentive systems. In Farazmand A(ed), Global encyclopedia of public administration, public policy, and governance. Springer Nature, Switzerland. 10.1007/978-3-319-31816-5_3706-1

[CR21] Coccia M (2019). Artificial intelligence technology in cancer imaging: Clinical challenges for detection of lung and breast cancer. J Soc Adm Sci.

[CR22] Coccia M (2021a) Pandemic prevention: lessons from COVID-19. Encyclopedia 2021, 1, 433–444. MDPI, Basel, Switzerland, Encyclopedia of COVID-19, open access journal. 10.3390/encyclopedia1020036

[CR23] Coccia M (2021). High health expenditures and low exposure of population to air pollution as critical factors that can reduce fatality rate in COVID-19 pandemic crisis: a global analysis. Environ Res.

[CR24] Coccia M (2021c) The relation between length of lockdown, numbers of infected people and deaths of COVID-19, and economic growth of countries: Lessons learned to cope with future pandemics similar to COVID-19 and to constrain the deterioration of economic system. Sci Total Environ 775:145801. 10.1016/j.scitotenv.2021.145801

[CR25] Coccia M (2021). Different effects of lockdown on public health and economy of countries: results from first wave of the COVID-19 pandemic. J Econ Lib.

[CR26] Coccia M, Farazmand A (2021). Comparative critical decisions in management. Global encyclopedia of public administration, public policy, and governance.

[CR27] Coccia M (2022). Meta-analysis to explain unknown causes of the origins of SARS-COV-2. Environ Res.

[CR28] Coccia M (2022). Preparedness of countries to face COVID-19 pandemic crisis: strategic positioning and underlying structural factors to support strategies of prevention of pandemic threats. Environ Res.

[CR29] Coccia M (2022). COVID-19 pandemic over 2020 (with lockdowns) and 2021 (with vaccinations): similar effects for seasonality and environmental factors. Environ Res.

[CR30] Coccia M (2022d) Improving preparedness for next pandemics: max level of COVID-19 vaccinations without social impositions to design effective health policy and avoid flawed democracies. Available online 31 May 2022. 113566. 10.1016/j.envres.2022.113566 10.1016/j.envres.2022.113566 PMC9155186 35660409

[CR31] Coccia M (2022). Optimal levels of vaccination to reduce COVID-19 infected individuals and deaths: a global analysis. Environ Res.

[CR32] Coccia M (2022f) The spread of the novel coronavirus disease-2019 in polluted cities: Environmental and demographic factors to control for the prevention of future pandemic diseases. In: Faghih N, Forouharfar A (eds) Socioeconomic dynamics of the COVID-19 crisis. Contributions to Economics. Springer, Cham, 351–369. 10.1007/978-3-030-89996-7_16

[CR33] Coccia M (2022g) Probability of discoveries between research fields to explain scientific and technological change. Technol Soc 68:101874. 10.1016/j.techsoc.2022.101874

[CR34] Coccia M, Bellitto M (2018). Human progress and its socioeconomic effects in society. J Econ Soc Thought.

[CR35] Coccia M, Finardi U (2013). New technological trajectories of non-thermal plasma technology in medicine. Int J Biomed Eng Techno.

[CR36] Coccia M, Rolfo S (2000) Ricerca pubblica e trasferimento tecnologico: il caso della regione Piemonte in Rolfo S. (eds) Innovazione e piccole imprese in Piemonte, FrancoAngeli Editore, Milano (Italy), pp. 236–256. ISBN: 9788846418784

[CR37] Goolsbee A, Syverson C (2021). Fear, lockdown, and diversion: comparing drivers of pandemic economic decline 2020. J Public Econ.

[CR38] Gupta V, Santosh KC, Arora R, (...), Kalid KS, Mohan S (2022) Socioeconomic impact due to COVID-19: an empirical assessment. Inf Process and Manag 59(2):102810 10.1016/j.ipm.2021.102810 PMC8829432 35165495

[CR39] Hale T, Angrist N, Goldszmidt R, Kira B, Petherick A, Phillips T, Webster S, Cameron-Blake E, Hallas L, Majumdar S, Tatlow H (2021). A global panel database of pandemic policies (Oxford COVID-19 Government Response Tracker). Nat Hum Behav.

[CR40] Homburg S (2020). Effectiveness of corona lockdowns: evidence for a number of countries. Econ Voice.

[CR41] Iyengar KP, Ish P, Botchu R (2022). Influence of the Peltzman effect on the recurrent COVID-19 waves in Europe. Postgrad Med J.

[CR42] Jamison JC, Bundy D, Jamison DT, Spitz J, Verguet S (2021). Comparing the impact on COVID-19 mortality of self-imposed behavior change and of government regulations across 13 countries. Health Serv Res.

[CR43] Johns Hopkins Center for System Science and Engineering, 2022. Coronavirus COVID-19 global cases, https://gisanddata.maps.arcgis.com/apps/opsdashboard/index.html#/bda7594740fd40299423467b48e9ecf6 (accessed in 4 March 2022)

[CR44] Khandia R, Singhal S, Alqahtani T, Kamal MA, El-Shall NA, Nainu F, Desingu PA, Dhama K (2022). Emergence of SARS-CoV-2 Omicron (B.1.1.529) variant, salient features, high global health concerns and strategies to counter it amid ongoing COVID-19 pandemic. Environ Res.

[CR45] Kim D, Lee YJ (2022). Vaccination strategies and transmission of COVID-19: evidence across advanced countries. J Health Econ.

[CR46] Kufel T, Kufel P, Błażejowski M (2022). Do COVID-19 lock-downs affect business cycle? Analysis using energy consumption cycle clock for selected European countries. Energies.

[CR47] Mahmoudi J, Xiong C (2022). How social distancing, mobility, and preventive policies affect COVID-19 outcomes: big data-driven evidence from the District of Columbia-Maryland-Virginia (DMV) megaregion. PLoS One.

[CR48] Meiry G, Alkaher D, Mintz Y, Eran Y, Kohn A, Kornblau G, Shneorson Z, Alkaher S, Sonkin R, Jaffe E (2022). The rapid development of AmboVent: a simple yet sustainable ventilation solution for use in a pandemic. Minim Invasive Ther Allied Technol.

[CR49] Nicoll A, Coulombier D. 2009. Europe’s initial experience with pandemic (H1N1) 2009—mitigation and delaying policies and practices. Euro Surveill 14(29): pii=19279 10.2807/ese.14.29.19279-en 19643049

[CR50] Núñez-Delgado A, Bontempi E, Coccia M, Kumar M, Farkas K, Domingo JL (2021). SARS-CoV-2 and other pathogenic microorganisms in the environment. Enviro Res.

[CR51] OECD Data (2022a) List of OECD Member countries, https://www.oecd.org/about/document/ratification-oecd-convention.htm (Accessed March 2022a)

[CR52] OECD Data (2022b) GDP, volume—annual growth rates in percentage. https://stats.oecd.org/index.aspx?queryid=60703 (Accessed March 2022b)

[CR53] Our World in Data (2022a) Coronavirus (COVID-19) vaccinations—statistics and research—our world in data https://ourworldindata.org/covid-vaccinations (Accessed 25 January 2022)

[CR54] Our World in Data (2022b) Ventilators (total number), variable time span: 2015–2020. https://ourworldindata.org/grapher/number-of-medical-ventilators

[CR55] Peltzman S (1975) The effects of automobile safety regulation. J Polit Econ 83(4):677–725. http://www.jstor.org/stable/1830396. Access 10 June 2022

[CR56] Prasad V, Jena AB (2014). The Peltzman effect and compensatory markers in medicine. Healthcare (Amsterdam, Netherlands).

[CR57] Pronti A, Coccia M (2021). Agroecological and conventional agricultural systems: comparative analysis of coffee farms in Brazil for sustainable development. Int J Sustain Dev.

[CR58] Qiu Z, Cao Z, Zou M, (...), Wang D, Du X (2022) The effectiveness of governmental nonpharmaceutical interventions against COVID-19 at controlling seasonal influenza transmission: an ecological study. BMC Infect Dis 22(1):331 10.1186/s12879-022-07317-2 PMC8977560 35379168

[CR59] Stringency Index (2022) COVID-19: stringency index (https://ourworldindata.org/covid-stringency-index, accessed February 2022)

[CR60] The World Bank (2022a) GDP per capita (constant 2010 US$). https://data.worldbank.org/indicator/NY.GDP.PCAP.KD (accessed March 2022).

[CR61] The World Bank (2022b) Data, population, total. https://data.worldbank.org/indicator/SP.POP.TOTL (Accessed January 2022).

[CR62] The World Bank (2022c) Current health expenditure (% of GDP), https://data.worldbank.org/indicator/SH.XPD.CHEX.GD.ZS (Accessed February 2022)

[CR63] Vinceti M, Filippini T, Rothman KJ, Di Federico S, Orsini N (2021). SARS-CoV-2 infection incidence during the first and second COVID-19 waves in Italy. Environ Res.

[CR64] WHO (2020) Estimating mortality from COVID-19, Scientific Brief. https://www.who.int/news-room/commentaries/detail/estimating-mortality-from-covid-19, 4 August (Accessed 6 May 2021)

[CR65] Wieland T (2020). A phenomenological approach to assessing the effectiveness of COVID-19 related nonpharmaceutical interventions in Germany. Saf Sci.

[CR66] Wilson N, Kvalsvig A, Barnard L (2020). Case-fatality risk estimates for COVID-19 calculated by using a lag time for fatality. Emerg Infect Dis.

[CR67] Wood SN (2021) Inferring UK COVID-19 fatal infection trajectories from daily mortality data: were infections already in decline before the UK lockdowns? Biometrics. https://doi.org/10.1111/biom.13462.doi:10.1111/biom.13462 10.1111/biom.13462 PMC8251436 33783826

[CR68] Yao L, Li M, Wan JY, (...), Bailey JE, Graff JC (2022) Democracy and case fatality rate of COVID-19 at early stage of pandemic: a multicountry study. Environ Sci Pollut Res 29(6):8694-8704 10.1007/s11356-021-16250-x PMC8421237 34490579

[CR69] Zhu P, Tan X (2022). Evaluating the effectiveness of Hong Kong’s border restriction policy in reducing COVID-19 infections. BMC Public Health.

